# Comparison of High-Intensity Interval Training and Moderate-to-Vigorous Continuous Training for Cardiometabolic Health and Exercise Enjoyment in Obese Young Women: A Randomized Controlled Trial

**DOI:** 10.1371/journal.pone.0158589

**Published:** 2016-07-01

**Authors:** Zhaowei Kong, Xitao Fan, Shengyan Sun, Lili Song, Qingde Shi, Jinlei Nie

**Affiliations:** 1 Faculty of Education, University of Macau, Macao, China; 2 Institute of Physical Education, Huzhou University, Huzhou, Zhejiang Province, China; 3 School of Physical Education and Sports, Macao Polytechnic Institute, Macao, China; University of Rome Foro Italico, ITALY

## Abstract

**Objective:**

The aim of this study was to compare the effects of 5-week high-intensity interval training (HIIT) and moderate-to-vigorous intensity continuous training (MVCT) on cardiometabolic health outcomes and enjoyment of exercise in obese young women.

**Methods:**

A randomized controlled experiment was conducted that involved thirty-one obese females (age range of 18–30) randomly assigned to either HIIT or MVCT five-week training programs. Participants in HIIT condition performed 20 min of repeated 8 s cycling interspersed with 12 s rest intervals, and those in MVCT condition cycled continuously for 40 min at 60–80% of peak oxygen consumption ($\dot{\text{V}}$O_2peak_), both for four days in a week. Outcomes such as $\dot{\text{V}}$O_2peak_, body composition estimated by bioimpedance analysis, blood lipids, and serum sexual hormones were measured at pre-and post-training. The scores of Physical Activity Enjoyment Scale (PAES) were collected during the intervention.

**Results:**

After training, $\dot{\text{V}}$O_2peak_ increased significantly for both training programs (9.1% in HIIT and 10.3% in MVCT) (*p =* 0.010, *η*^*2*^ = 0.41). Although MVCT group had a significant reduction in total body weight (TBW, −1.8%, *p* = 0.034), fat mass (FM, - 4.7%, *p* = 0.002) and percentage body fat (PBF, −2.9%, *p* = 0.016), there were no significant between-group differences in the change of the pre- and post-measures of these variables. The HIIT group had a higher score on PAES than the MVCT group during the intervention. For both conditions, exercise training led to a decline in resting testosterone and estradiol levels, but had no significant effect on blood lipids.

**Conclusion:**

Both HIIT and MVCT are effective in improving cardiorespiratory fitness and in reducing sexual hormones in obese young women; however, HIIT is a more enjoyable and time-efficient strategy. The mild-HIIT protocol seems to be useful for at least maintaining the body weight among sedentary individuals.

## Introduction

Overweight and obesity has become a serious public health problem worldwide. Currently, more than 35% of men and close to 40% of women are overweight or obese [1]. Although weight increase occurs for all ages in both developed and developing countries, such increase is the most rapid for people between 20 and 40 years of age [2]. Overweight and obesity are associated with lower level of life quality [3], and with cardiovascular disease, hyperlipidemia [4] and cancer [5], while exercise-induced weight reduction improves cardiorespiratory fitness [6] and lowers metabolic risk factors [6, 7]. The recommended exercise prescription for most adults is typically a regular moderate-intensity continuous exercise program, with ≥30 min·d^−1^ on ≥5 d·wk^−1^ for a total of ≥150 min·wk^−1^ or an alternative of vigorous exercise for 75 min or more per week, for improving and maintaining physical fitness and health [7]. However, the inactive nature of sedentary individuals and the “lack of time” excuse tend to prevent people from being engaged in regular physical activity [8].

Low cardiorespiratory fitness level is the most powerful predictor for developing cardiovascular disease and the mortality induced by such disease [9]. An increasing amount of evidence indicates that high-intensity intermittent/interval exercise training (HIIT) has positive effects on cardiorespiratory fitness, obesity and the associated comorbidities [3, 10]. Although some studies reported that protocols as short as two weeks in duration have resulted in marked increments in $\dot{\text{V}}$O_2max_ by 9% to 13% [10, 11], the results of interventions with durations ranging from 2 to 6 weeks are inconsistent [12, 13].

Overweight and obesity may affect women’s health with irregular menstrual cycle and abnormal sex hormone levels [5], as high levels of androgen and estradiol are observed in obese young women [14]. Sex steroids hormones are involved in the preservation and expansion of muscle mass [15], and are associated with the regulation of body fat distribution in both men and women [14]. Androgen and estrogen have similar effects on lipid metabolism in that higher levels of androgen and estrogen may increase lipolysis and may promote lipid oxidation in animals [16] and in vitro [17]. Therefore, sex steroid hormones may play a supportive role in body weight change.

Because the Wingate-based sprint interval training is exceptionally demanding, less rigorous HIIT protocols have been developed for the sedentary individuals [18]. It has been reported that training with 8s/12s work-rest intervals for 20 minutes for 15 weeks improved body fat distribution and insulin resistance of young women, when compared to 40 min of steady-state exercise with the similar energy expenditure [3]. Nevertheless, HIIT is typically characterized as a low-volume training program [19, 20], and theoretically, for the purpose of examining health benefits, there is no requirement that this exercise program should have the same energy expenditure as the continuous training program. Additionally, in terms of the study designs for fat loss induced by HIIT, there are some common flaws, such as the lack of quantitative estimation of energy expenditure [21–24], and the neglect of the effects of other physical activities of the participants besides the implemented exercise training program [3, 22–24].

Enjoyment is an important factor for long-term adherence. A constructed exercise program should be perceived as enjoyable and be time efficient. If not, it is difficult to sustain the exercise long enough to get the desired health outcomes. Although it has been reported that HIIT seems to be more enjoyable than either continuous moderate-intensity exercise [25] or continuous vigorous-intensity exercise (MVCT) [26], Foster et al (2015) reported that 8 weeks of the Tabata protocol consisting of 20 s cycling at 170% $\dot{\text{V}}$O_2max_ with 10 s rest for eight sets was less enjoyable than continuous training protocol of 20 minutes with an intensity at 90% ventilatory threshold among untrained young adults [27]. As a consequence, there has been the argument against the use of vigorous exercise as an alternative to traditional continuous exercise: vigorous exercise is harder, and thus it could be a deterrent for the sedentary/obese population [8, 28]. Given that the current findings were based on samples of young active men [25] or normal weight individuals [26], the question concerning whether the low-volume HIIT training is more enjoyable than continuous training needs to be examined further in different populations, especially in overweight and obese subjects.

At present, it is not clear if HIIT has any additive physiological effects relative to MVCT. Up to this time, in the studies that compared continuous exercise with HIIT in overweight and obese individuals, the continuous exercise protocols typically had low to moderate exercise intensity [20]. Given that, with the same exercise volume, higher exercise intensity is more effective for improving aerobic capacity than lower exercise intensity [29], it is necessary to use a higher-intensity exercise as the reference group in the comparison of the physiological differences between continuous training and HIIT. Few studies have examined the health related outcomes between continuous training with moderate-to-vigorous intensity around 150 minutes per week and HIIT training for obese women, despite the fact that MVCT training has been used for athletes [30], active individuals [31, 32], and sedentary individuals with normal body weight [33]. Thus, whether HIIT exercise protocols would result in different effects on cardiorespiratory fitness and metabolic indices when compared to MVCT exercise protocols among overweight and obese individuals warrants further investigation.

Using a less demanding HIIT protocol with 60 repetitions of 8s/12s work-rest intervals at a lower resistance for 4 days/week, the purpose of this study was to compare the effects of 5-week HIIT and MVCT interventions on cardio-metabolic health outcomes, sex hormones levels and physical activity enjoyment scale (PAES) in obese female subjects. It was hypothesized that both HIIT and MVCT would lead to improvement in cardio-metabolic parameters, including $\dot{\text{V}}$O_2peak_, body weight and fat, blood lipids, and sexual hormones, and that different training modes would have different effects on these health-related outcomes. Additionally, it was hypothesized that the time-efficient HIIT would be a more enjoyable exercise mode in comparison with MVCT.

## Method

### Subjects

A power analysis was conducted by G*Power Version 3.1 to justify the sample size. With a power of 0.8 at α  =  0.05, an assumed correlation of 0.8 between pre-treatment and post-treatment, and an effect size of 0.32 based on a meta-analysis for the primary outcome of $\dot{\text{V}}$O_2max_ resulting from high-intensity interval training [19], the sample size for HIIT group is estimated to be 12. When a dropout rate of 20% is allowed, a total sample size of 30 subjects is needed.

**Fig 1:**
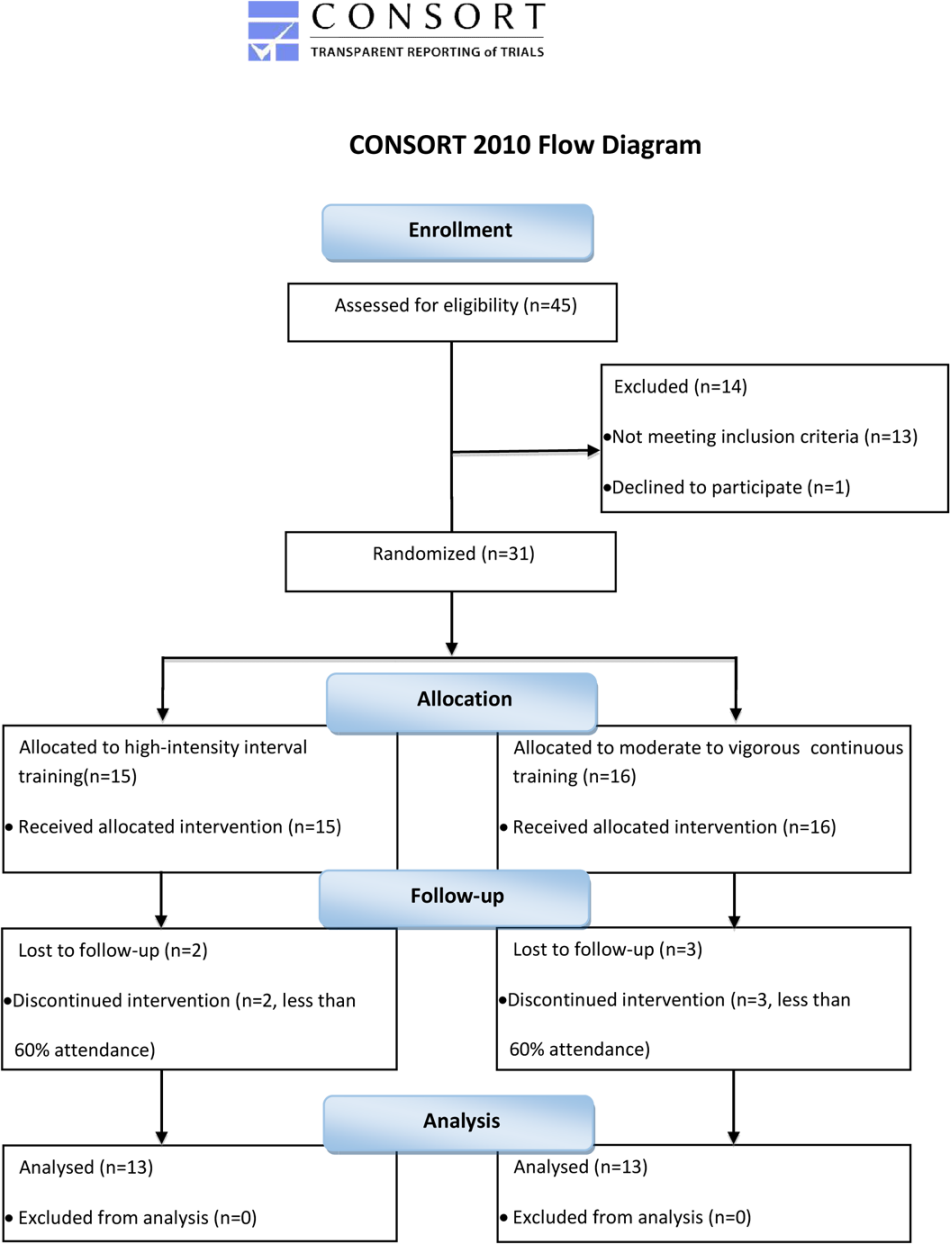
Flow-chart of the study.

Volunteers were publicly recruited through local advertisements to participate in the study (Consort Flow Diagram, Fig 1), which was approved by the Panel on Research Ethics of the University of Macau (S1 Protocol).

The inclusion criteria for the subjects were: in the age range of 18 to 35; being “sedentary” as defined by reporting less than 60 minutes exercise every week in the previous 6 months; being “obese” as defined as the percentage of body fat over 30% [34] measured by a 5-serial of frequent bioimpedance analyzer (Biospace Inbody 720, South Korea), and with a doctor certificate approving the practice of vigorous exercise. Each volunteer completed a PAR-Q form and a menstrual cycle survey prior to being admitted into the study. Volunteers who could not participate in strenuous physical exercise, smokers, alcoholics, and users of contraceptive pills or prescribed drugs were excluded. A total of 31 qualified subjects were included. All subjects provided written informed consent before participation, and then subjects were randomly assigned to either HIIT or MVCT group. By the end of the study, twenty-six subjects completed all the testing procedure and training sessions.

### Experimental protocol

The experimental protocol consisted of pre- and post-training measurements and a 5-week HIIT or MVCT intervention. Measures of $\dot{\text{V}}$O_2peak_, body composition and blood assays were taken in pre- and post-training measurements. Baseline measures were taken within 96 h and 144 h before the 5-week exercise intervention, and post-training measures were taken between 72 h and 120 h following the last training session. For each subject, both pre- and post-training measures were taken either in the follicular phase or in the late luteal phase, based on the self-reported menstrual cycle survey. During both the testing and training sessions, the same verbal encouragement was given by the same assistants.

### Blood sampling

Blood samples were taken from the cubital veins at pre- and post-training after a minimum of a 12 h overnight fast. After clotting for 1 hour at room temperature, the samples were centrifuged at 3000 rpm for 10 min and then serum supernatants were removed and frozen at– 80°C for later analysis.

### Body composition analysis

Height and weight were determined using standard conventional methods (in light clothing and no footwear) with a stadiometer and an electronic scale respectively, and body mass index (BMI, in kg/m^2^) was calculated by weight divided by square height. Body composition was assessed in a fasting state at baseline and approximately 72 h following the last training session.

### $\dot{\text{V}}$O_2peak_ test

$\dot{\text{V}}$O_2peak_ test was carried out on a computer-controlled ergometer (Monark 839E, Sweden) both at baseline and at least 72 h following the last training session. After a 2 min cycling warm-up at 30 W, graded exercise started at the initial workload of 50 W and increased by 25 W every 3 min till completion. Each subject was asked to maintain the cycling speed of 60 ± 5 rpm until she either reached the criteria described below or reached the stage of volitional exhaustion. $\dot{\text{V}}$O_2peak_ was assessed continuously using a pre-calibrated breath-by-breath analysis system (Meta-Max 3B, Cortex Biophysik GmbH, Leipzig, Germany). The test terminated when the subjects met any two of the following criteria: $\dot{\text{V}}$O_2_ reached a plateau with a change less than 150 mL·min^−1^; heart rate reached age-predicted maximal level (220-age); respiratory exchange ratio (RER) ≥ 1.10, and rating of perceived exertion (Borg’s) scale reached 18 [21]. After the test, there was a 5-min recovery at 30 W $\dot{\text{V}}$O_2peak_ was determined as the highest average $\dot{\text{V}}$O_2_ value maintained for one minute.

### High-intensity interval exercise training protocol

The HIIT cycling protocol was similar to that used in a previous study by Trapp et al [3]. Briefly, after a 3-min warm-up at 50 W, each participant followed a prerecorded tape to conduct 8 s of sprinting and 12 s of passive rest for a maximum of 60 repetitions on an ergometer (Monark 874E, Sweden). The initial resistance was 1.0 kg and subjects worked as hard as they could during the sprinting phase. The resistance would be increased by 0.5 kg increment once an individual could complete two consecutive 20-min intermittent sprinting exercise sessions at the given intensity level. Subjects reduced their workload at the end of this conditioning phase and cooled down for 3 min at 50 W followed by standard stretches. Heart rate (HR, Polar F4M BLK, Finland), the Borg 6–20 ratings of perceived exertion (RPE) and training power were recorded before and after every five sprints, and enjoyment of exercise was assessed by PAES scale [35] immediately after every training session. When an individual missed a session, she would be asked to make it up later so that 20 HIIT sessions were completed within 5 weeks.

### Moderate-to-vigorous intensity continuous exercise training protocol

For MVCT training [3], each subject seated on an ergocycle (Ergometer 900PC, Ergoline, Germany) to start a 3-min warm-up at 50 W, and continued to exercise at an initial workload of 60% $\dot{\text{V}}$O_2peak_ of the pre-training test for 40 min with a rhythm at 60 ± 5 rpm. After every training session, similar to the HIIT group, there was a 3-min cool-down and stretching period. Once an individual had completed two consecutive exercise sessions at the specified level of exercise intensity, resistance was increased by 0.5 kg until she reached the level of 80% $\dot{\text{V}}$O_2peak_ of the pre-training test. HR, RPE and training power were recorded every 5 min during the exercise training, PAES scores were assessed in the same way as for the HIIT group.

### Training intensity and energy expenditure during training

In terms of training intensity, the actual values of intensity were the mean of $\dot{\text{V}}$O_2_ measured in the first and last session in HIIT group, and the average value of $\dot{\text{V}}$O_2_ determined in all 20 sessions in MVCT group, respectively. The exercise intensity in all subjects of MVCT reached the required 80% $\dot{\text{V}}$O_2peak_ after two weeks of exercise training. Energy expenditure of HIIT was assessed as the mean of these two sessions, and the data was used to evaluate energy expenditure for the 5 weeks of HIIT, while energy expenditure for the MVCT group was estimated by converting the workload for each session into oxygen consumption [3, 36].

### Record of diet and extra physical activity

In order to ensure that the effect of the exercise intervention was not counteracted in some way by other co-concurring events, subjects were instructed to maintain their normal diet and normal daily physical activities throughout the training program. Food intake data were recorded by using a 3-day diet recall protocol (two weekdays and one weekend day) during three weeks: one week before the intervention, the third week of the intervention, and the last week of the intervention. Energy intake and diet components were analyzed by using the nutrition analysis and management system for athletes and the general public (NRISM Version 3.1), and the analyses were conducted by the Sports Nutrition Research Center, National Institute of Sports Medicine, China. Daily physical activities were monitored by using a pedometer (Yamax SW-200 digiwalker, Japan) on three days (two weekdays and one weekend day) in every week during the 5-week training period.

### Measures of blood lipids and sexual hormones

Serum lipids, including high-density lipoprotein cholesterol (HDL-C), low-density lipoprotein cholesterol (LDL-C), total cholesterol (TC) and total triglyceride (TG), were measured by using an automatic biochemical analyzer (Olympus AU400, Japan). The intra-assay coefficients of variation (CV) for blood lipid assays were all within 5%. Concentrations of serum testosterone and estradiol were analyzed by using the commercially available electrochemiluminescence immunoassay kits (Roche Diagnostics GmbH, Mannheim, Germany). The lower detection limits and intra-assay CVs were 0.025 ng·ml^-1^ and 4.5% for testosterone, and 5 pg·ml^-1^ and 4.8% for estradiol. All analyses were tested in the same assay with standard procedures (Deyi Biomedical Technology Co., Ltd., Beijing, China).

### Statistical analysis

Data analysis was conducted by PASW software (Release 22.0; IBM, New York, USA). Prior to the planned statistical analyses, preliminary analysis was conducted (Kolmogorov-Smirnov test) to confirm data distribution normality. Once it was confirmed that the sample data satisfied the normality assumption, statistical analyses relevant for our main research interest were conducted. A two-way mixed analysis of variance (ANOVA) with repeated measures was used to test the main effect (i.e., group effect) and the interaction effect (time and group interaction) on the outcome variables. For within–group change from pre- and post-values of the relevant outcome variables of interest, paired-sample *t*-test was performed to test the difference in pre- and post-measures. Change between the pre- and post-measures was calculated for each outcome variable of interest. Once the effect of intervention was shown to be statistically significant, ANCOVA with the baseline value as covariate was performed to determine the difference of the change between the two groups. Pearson-product moment correlation coefficients were computed to examine the relationships between the cardio-metabolic health outcomes and sexual hormones. As effect size measure of the main effect and the interaction effect, *partial η*^*2*^ was considered small if *η*^*2*^<0.06, and large if *η*^*2*^ >0.14 [37]. All experimental data are presented as means ± standard deviation (SD). A *p* value of < 0.05 was used as the criterion for statistical significance.

## Results

### Training data

Weekly training time spent in HIIT was less than half of the time in MVCT (66 ± 4 min in HIIT group vs. 148 ± 12 min in MVCT group (*p* = 0.000), and the former had lower RPE than the latter (*p =* 0.035). The ratio of training HR and HRmax as determined at $\dot{\text{V}}$O_2peak_ test in HIIT tended to be higher than that of MVCT (*p =* 0.072), despite similar training HR in the two intervention conditions. In terms of %$\dot{\text{V}}$O_2peak_ during the training sessions, HIIT group maintained a mean level of 80 ± 7% $\dot{\text{V}}$O_2peak_ while MVCT group maintained the level of 71 ± 8% $\dot{\text{V}}$O_2peak_ (*p* = 0.000). Over the 5-week training period, the total estimated energy expenditure in MVCT group was twice as much as that in HIIT group (*p* = 0.000) (Table 1).

**Table 1:** Training data during intervention.

	HIIT (n = 13)	MVCT (n = 13)	*p*
Weekly training time (min)	66 ± 4	148 ± 12	0.000
Total training time (min)	332 ± 21	740 ± 58	0.000
Workload (w)	214 ± 37	114 ± 17	0.000
Intensity (%$\dot{\text{V}}$O_2peak_)	80 ± 7*	71 ± 8	0.000
HR (bpm)	162 ± 8	158 ± 12	0.009
HR/HRmax (%)	86 ± 4	84 ± 4	0.072
RPE	14 ± 1	15 ± 1	0.035
Total exercise energy expenditure (KJ)	12919 ± 2159	26125 ± 2986	0.000
Total exercise energy expenditure (Kcal)	3088 ± 516	6244 ± 714	0.000

Observed values are expressed as means ± standard deviation. *p* (values) for comparisons of variables during intervention. HIIT: High-intensity interval training, MVCT: moderate-to-vigorous continuous training. $\dot{\text{V}}$O_2peak_: peak oxygen uptake.

*Intensity was the mean of %$\dot{\text{V}}$O_2peak_ measured in the first and last training session.

### Diet and extra physical activities

**Fig 2:**
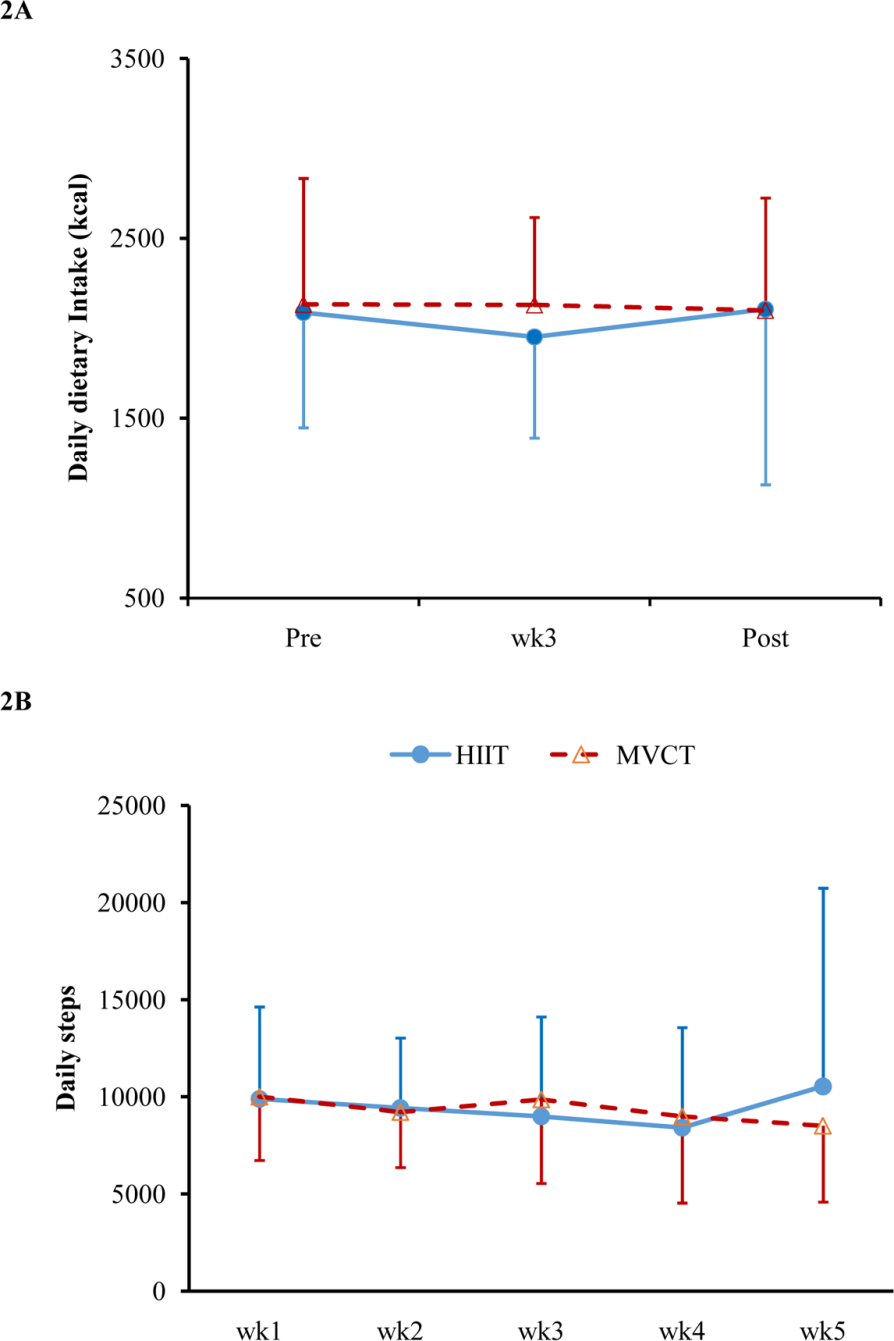
**Daily dietary intake (2A) and steps recorded by pedometers (2B) during the intervention.** There were no significant differences between HIIT and MVCT group.

There were no within- and between-group differences in daily energy intake and physical activity at pre-, during-, and post-intervention times (*p* > 0.05) (Daily energy intake and physical activity, Fig 2). The proportions of macronutrient intake were approximate 50%, 35% and 15% in carbohydrate, fat and protein in both groups.

### Cardiorespiratory fitness (measured in $\dot{\text{V}}$O_2peak_)

Both exercise modalities improved $\dot{\text{V}}$O_2peak_ after training (*p =* 0.010, *η*^*2*^ = 0.41). HIIT group improved by 9.1% (*p =* 0.046) and MVCT group by 10.3% (*p =* 0.004), respectively. The improvement difference between the two groups was not statistically significant.

### Body composition

There were statistically significant decreases on weight (*p* = 0.034), BMI (*p* = 0.034), FM (*p* = 0.002) and PBF (*p* = 0.016) in the MVCT group after the intervention. In contrast, no statistical changes in these body composition measures were found in HIIT group (*p* > 0.05). When adjusted for baseline values, there were no between-group differences in body composition (Tables 2 and 3).

**Table 2:** Outcome measures before and after five weeks of exercise training.

	HIIT (n = 13)		MVCT (n = 13)		Within group		Interaction effect	
	Pre	Post	Pre	Post	*p*	*Partial η*^*2*^	*p*	*Partial η*^*2*^
Age (y)	21.5 ± 4.0		20.5 ± 1.9					
Weight (kg)	69.1 ± 9.5	69.7 ± 9.3	67.5 ± 7.3	66.3 ± 6.6	0.530	0.04	0.010	0.25
BMI (kg·m^-2^)	25.8 ± 2.6	26.0 ± 2.5	25.5 ± 2.1	25.0 ± 2.0	0.352	0.04	0.009	0.25
MM (kg)	24.3 ± 2.6	24.6 ± 2.7	23.8 ± 2.4	23.8 ± 2.0	0.409	0.03	0.437	0.03
FM (kg)	24.6 ± 5.9	24.9 ± 5.4	24.0 ± 4.1	22.8 ± 3.6	0.124	0.10	0.012	0.23
PBF (%)	35.2 ± 4.0	35.4 ± 3.4	35.4 ± 3.3	34.2 ± 2.4	0.162	0.08	0.053	0.15
TC (mmol·L^-1^)	4.3 ± 0.8	4.3 ± 0.5	4.3 ± 0.5	4.3 ± 0.6	0.990	0.00	0.994	0.00
HDL-C (mmol·L^-1^)	1.3 ± 0.2	1.4 ± 0.2	1.3 ± 0.2	1.3 ± 0.2	0.811	0.00	0.255	0.05
LDL-C (mmol·L^-1^)	2.4 ± 0.8	2.3 ± 0.4	2.4 ± 0.5	2.4 ± 0.6	0.921	0.00	0.825	0.00
TG (mmol·L^-1^)	1.0 ± 0.5	0.9 ± 0.5	0.9 ± 0.3	1.0 ± 0.4	0.886	0.00	0.418	0.03
Testosterone (ng·dl^-1^)	14.2 ± 5.5	11.5 ± 3.6	11.2 ± 4.2	9.5 ± 2.9	0.071	0.14	0.665	0.01
Estradiol (pg·ml^-1^)	154.8 ± 129.8	76.9 ± 51.6	161.2 ± 166.5	97.6 ± 78.6	0.035	0.19	0.882	0.00
$\dot{\text{V}}$O_2peak_ (ml·min^-1^·kg^-1^)	32.0 ± 6.6	34.3 ± 7.5	32.0 ± 5.0	35.8 ± 6.9	0.000	0.41	0.307	0.04

Observed values are expressed as means ± standard deviation. *p* (values) for within-group (time) effect and interaction (time × group) effect. Partial *η*^*2*^ value for effect size (ES).

HIIT: High-intensity interval training, MVCT: moderate-to-vigorous continuous training, BMI: body mass index, MM: muscle mass, FM: fat mass, PBF: percentage of body fatness, TC: total cholesterol, HDL-C: high-density lipoprotein cholesterol, LDL-C: high-density lipoprotein cholesterol, TG: triglycerides, $\dot{\text{V}}$O_2peak_: peak oxygen uptake.

**Table 3:** Changes in outcome measures after intervention.

	HIIT (n = 13)		MVCT (n = 13)		Between-group Difference
	Δ	*p* ^*a*^	Δ	*p* ^*a*^	*p* ^*b*^
Weight (kg)	0.6 ± 1.5	0.163	-1.3 ± 1.9	0.034	0.397
BMI (kg·m^-2^)	0.2 ± 0.5	0.163	-0.5 ± 0.7	0.034	0.963
MM (kg)	0.3 ± 0.6	0.152	0.0 ± 0.9	0.977	0.063
FM (kg)	0.3 ± 2.6	0.514	-1.2 ± 1.1	0.002	0.811
PBF (%)	0.2 ± 3.5	0.713	-1.1 ± 1.4	0.016	0.733
TC (mmol·L^-1^)	0.0 ± 0.9	0.998	0.0 ± 0.7	0.988	0.897
HDL-C (mmol·L^-1^)	0.0 ± 0.1	0.254	0.0 ± 0.2	0.579	0.648
LDL-C (mmol·L^-1^)	0.0 ± 0.8	0.839	0.0 ± 0.6	0.922	0.576
TG (mmol·L^-1^)	-0.1 ± 0.4	0.457	0.1 ± 0.4	0.669	0.240
Testosterone (ng·dl^-1^)	-2.7 ± 6.2	0.285	-1.7 ± 5.1	0.507	0.818
Estradiol (pg·ml^-1^)	-77.9 ± 138.0	0.276	-63.5 ± 168.6	0.565	0.871
$\dot{\text{V}}$O_2peak_ (ml·min^-1^·kg^-1^)	2.3 ± 3.7	0.046	3.8 ± 3.9	0.004	0.701

Observed values are expressed as means ± standard deviation. Delta (Δ) change from baseline to post-intervention. *p* ^*a*^ (values) for the difference in pre-and post-measures, and *p* ^*b*^ (values) for between-group comparisons in changes adjusted for baseline values.

HIIT: high-intensity interval training, MVCT: moderate-to-vigorous continuous training, BMI: body mass index, MM: muscle mass, FM: fat mass, PBF: percentage of body fatness, TC: total cholesterol, HDL-C: high-density lipoprotein cholesterol, LDL-C: high-density lipoprotein cholesterol, TG: triglycerides, $\dot{\text{V}}$O_2peak_: peak oxygen uptake.

### Blood lipids and sexual hormones

There were no significant differences in blood lipid profiles between pre- and post-training measures (*p* > 0.05). Serum testosterone tended to be significantly lower (*p* = 0.071, *η*^*2*^ = 0.14) and estrogen decreased significantly (*p* = 0.035, *η*^*2*^ = 0.19) after training intervention (Table 2). No group differences were found in these two sexual hormones (Table 3).

### Physical activity enjoyment scale (PAES) during the intervention

**Fig 3:**
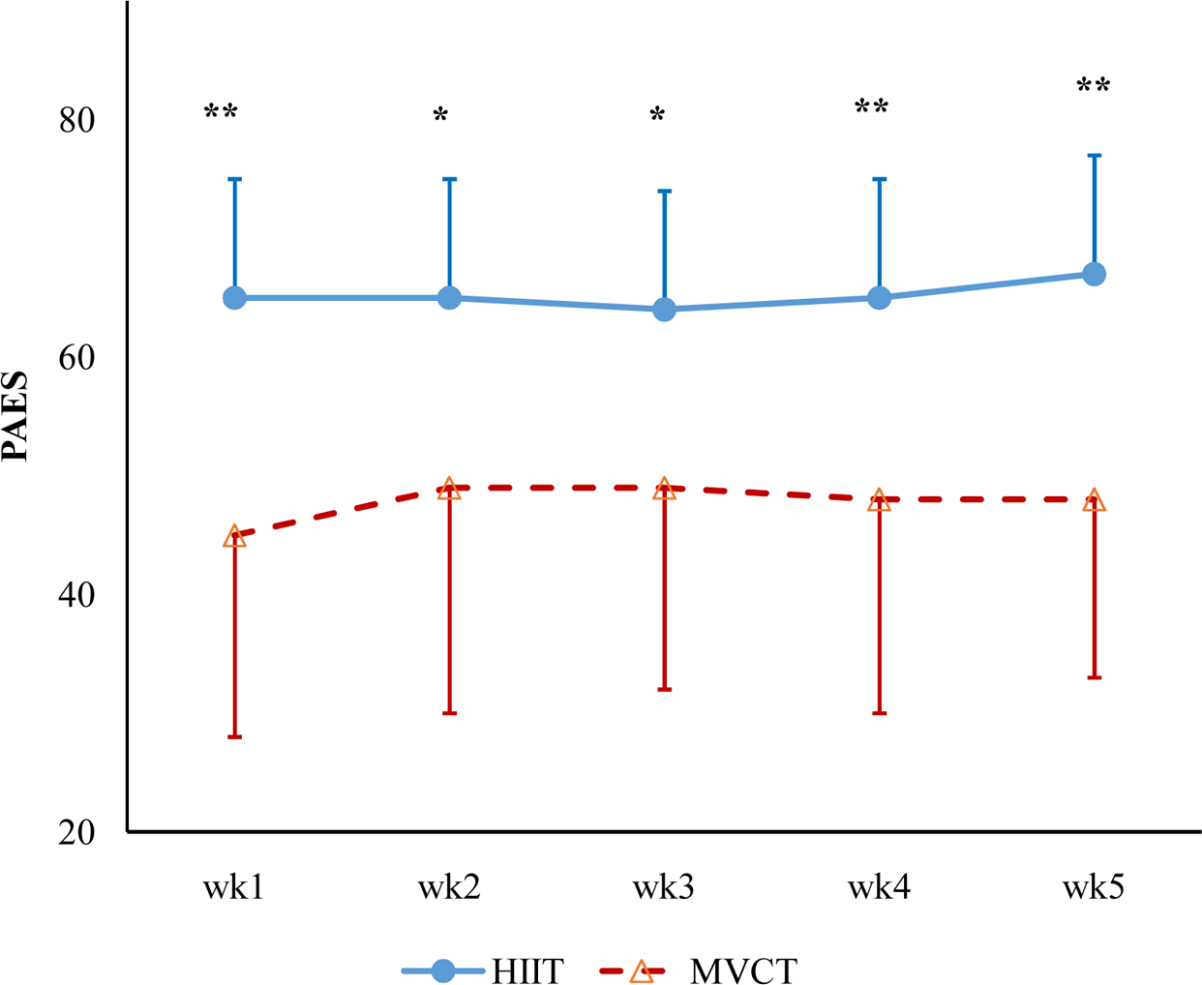
Physical activity enjoyment scale (PAES) during the intervention. HIIT had significant higher scores than those of MVCT in any week of exercise intervention. *p < 0.05, **p < 0.01.

HIIT group had significantly higher scores on PAES than the MVCT group in any of the 5 weeks during the exercise intervention (Physical activity enjoyment scale, Fig 3).

### Correlations between variables

No significant correlations were found between the variables among $\dot{\text{V}}$O_2peak_, body composition, blood lipids and sexual hormones. However, there were moderate correlations between the total energy expenditure and body weight (*r* = -0.55, *p =* 0.005), BMI (*r* = -0.55, *p* = 0.005), fat mass (*r* = -0.54, *p* = 0.007) and PBF (*r* = -0.43, *p* = 0.038) in all subjects.

## Discussion

The present study showed that both HIIT and MVCT training interventions (20 sessions/week for 5 weeks) enhanced $\dot{\text{V}}$O_2peak_ significantly, and the training modes had no difference on body composition, even though MVCT resulted in a significant reduction in body weight, BMI and FM. On the other hand, subjects in HIIT condition had higher PAES scores during training intervention, and they used half of the exercise time to achieve similar improvement in $\dot{\text{V}}$O_2peak_. The findings here suggest that both short-term HIIT and MVCT improve cardiorespiratory fitness, however HIIT is more time-efficient and enjoyable for the subjects.

We observed that the HIIT and MVCT interventions led to a similar improvement in $\dot{\text{V}}$O_2peak_ by ~10% in obese young women, indicating that the low-volume HIIT protocol (8s sprinting interspersed with 12s rest) employed in the present study, which is about half the time and half of the energy expenditure of the continuous exercise practice, is a time-efficient means for improving cardiorespiratory fitness in overweight and obese young women. As indicated in the research literature, the rapid improvement in $\dot{\text{V}}$O_2max_ or $\dot{\text{V}}$O_2peak_ after 2–8 weeks of HIIT training may be the result of enhanced central functions such as an increment in stroke volume [38], and/or the result of peripheral adaptation of skeleton muscles by up-regulating various mitochondrial enzymes activities such as citrate synthase [32], pyruvate dehydrogenase [10], 3-hydroxyacyl CoA dehydrogenase [11, 32] and cytochrome oxidase [39]. It is generally acknowledged that low cardiovascular fitness is a good predictor of heart disease. According to the dose-response analyses, each 1-MET improvement in cardiorespiratory fitness was associated with 15% decrement in risk of cardiovascular disease [40]. The magnitudes of the improvement in $\dot{\text{V}}$O_2peak_ as a result of the prescribed exercises were 0.7 ± 0.3 MET in HIIT and 1.1 ± 0.3 MET in MVCT, which seem to be effective in reducing the risk of cardiovascular diseases [23, 41].

Inconsistent with our hypothesis, HIIT did not lead to any beneficial changes in weight loss and fat loss in this study. This finding, however, is in agreement with some previous studies that implemented very demanding protocols for 6 weeks [32], and even for 12 weeks of HIIT intervention [42]. But our finding differs from that reported in Trapp et al’s study [3], which showed that similar HIIT protocol for a longer duration (15 weeks, as compared to 5 weeks in our study) led to significant improvement in body composition in a different population (healthy subjects, as compared to obese young women in the present study). The discrepancy could be the result of the lengths of the intervention and the characteristics of the subjects. Although the MVCT intervention led to significant reductions in weight, BMI and FM, when adjusted with baseline values, no group differences between HIIT and MVCT were found in changes of body composition. Given the similar energy intakes and the other daily physical activities between the two groups, the different exercise energy expenditure levels (26125 ± 2986 KJ in the MVCT group vs. 12919 ± 2159 KJ in the HIIT group) are most likely to have resulted in the similar outcomes on weight and fat loss.

While total exercise energy expenditure has explained 18–30% of the reductions of body weight, BMI, FM and PBF, the lack of group differences suggests that the initial FM could be significantly related to fat loss [3]. Thus, although HIIT intervention with a longer duration may improve body composition [3, 24], at this time, there is no conclusive evidence that low-volume mild HIIT would have more favorable effects than high-volume MVCT on weight loss and body fat loss in overweight and obese populations [42]. Additionally, given the progressive increase of obesity prevalence [1], and given that the improvement of health-related outcomes could be independent of weight loss [6, 7], maintaining the body weight and not to have further weight gain in overweight and obese individuals under HIIT exercise condition could be considered as beneficial.

As well-established metabolic risk factors [22], blood lipids are associated with cardiovascular disease [23]. However, we found no positive changes of blood lipids in both HIIT and MVCT conditions among the obese young women. Almost no studies reported that 2–16 weeks of HIIT resulted in improvement of TC, LDL-C and TG [22, 43, 44], while a positive finding for HDL-C has been reported [43]. Based on what is known now that a weekly minimum exercise energy expenditure of 1200 to 2200 kcal is the necessary minimum to yield positive alteration in blood lipids [4], the effect of any exercise on blood lipids may not be observable until certain exercise thresholds are met [4]. Consequently, with insufficient weekly exercise caloric expenditure (1249 ± 143 kcal/wk in MVCT group and 628 ± 105 kcal/wk in HIIT group) in a short-term intervention, it is not surprising that the present study did not show any favorable changes in blood lipids in obese females. A recent study, however, contradicted this expectation, as it showed that 20 min HIIT training for 18 sessions in 6 weeks significantly decreased TC, TG, medium VLDL-C and medium HDL-C in overweight or obese males [23]. It appears that further investigations are needed to examine the efficacy of different HIIT protocols on blood lipids, especially taking lipoprotein subclasses into account [23]. However, in designing such studies in the future, it is important to control for the lipid-altering confounding factors, such as body weight, fat mass, caloric intake, nutrient composition of diets, and other potentially lipid-altering lifestyle characteristics [4].

After 5 weeks of exercise training, serum testosterone and estradiol decreased in all obese young women in the present study, suggesting that short-term exercise training could result in decreased circulating levels of testosterone and estradiol in obese young females. However, the changes of these steroid hormones were not associated with the improvement of cardiorespiratory and weight loss. Previous studies showed that, even for eumenorrheic healthy young women with normal weight, in response to resistance training [45] or aerobic training [46, 47], resting testosterone and estradiol levels showed inconsistent changes, including decrease [47], no change [46] or increase [45]. Inconsistent with the present study, Almenning et al. (2015) found that high-intensity interval training had no significant effect on serum testosterone, despite improvement in cardiometabolic profile [48] in women with polycystic ovary syndrome (PCOS). Given that sampling in different menstrual phases may have possible influence on sex hormone concentrations, we also re-examined the data by only including those participants whose pre- and post-training blood samples were taken at the same phase of the menstrual cycle, that is, for each subject, both pre- and post-training samples were taken either at her follicular phase, or at her luteal phase. We removed the subjects whose pre- and post-training samples were not taken at the same phase (e.g., one at the follicular phase and the other at the late luteal phase) of the menstrual cycle. This re-analysis excluded 6 subjects in HIIT group, and 7 in MVCT group. After the removal of these subjects from the analysis, similar reductions were still observed in circulating testosterone (*p* = 0.090, *η*^*2*^ = 0.24) and estradiol (*p* = 0.089, *η*^*2*^ = 0.24). Nevertheless, we could not rule out the possibility that the changes in steroid hormones might be due to some factors un-related to our exercise training, because this study did not have a control group of no-exercise. Interestingly, the differences in the reduction of resting testosterone and estradiol levels in obese young women between HIIT and MVCT interventions were small, suggesting that exercise intensity may not have significant effect on serum testosterone and estradiol in overweight and obese individuals.

Affective response to exercise is one important feature of the exercise experience [49]. Besides motivation and competence, feelings of pleasure and enjoyment can predict the adherence to exercise [50]. People tend to avoid participating in exercise if they find it too strenuous, particularly those from sedentary populations [8]. Therefore, it was suspected that HIIT would not be considered enjoyable by the sedentary overweight and obese subjects in this study, although Bartlett et al [25] showed that young, lean and fit men had higher PAES scores after a HIIT running protocol than after a continuous running protocol. In the present study, the PAES scores of enjoyment of exercise were taken immediately after every training session, and this was different from the study by Bartlett et al. [25] in which PAES scores were taken during cool-down period after the high-intensity intermittent exercise. Moreover, the PAES scores used in the analyses in this study were the average PAES scores across four training sessions each week. We observed that PAES scores in HIIT were significantly higher than those measured in MVCT condition during the intervention, contrary to the findings reported in some previous studies [49], and to the finding of lower adherence in HIIT reported before [51]. The seemingly conflicting results could be the result of different HIIT protocols concerning exercise intensity and duration in different studies, and differences in factors such as timing of the work-recovery cycles, type, intensity and number of repetitions, etc. [42, 52]. In the present study, we selected a relatively mild HIIT protocol for inactive obese young women, and the subjects in this HIIT protocol showed similar mean HR, and even lower RPE, as those in the continuous exercise protocol. Furthermore, when the PAES scores in the first two training sessions were compared, the continuous training group still had significantly lower scores than those of the HIIT group (65 ± 10 in HIIT group and 45 ± 17 in MVCT group, *p* = 0.001). This finding, as partially supported by the previous studies [25], indicates that HIIT is also more enjoyable than moderate-intensity continuous training in overweight and obese individuals. Collectively, such information suggests that the present HIIT protocol was readily acceptable by sedentary obese young women, and thus could be a more enjoyable and time-efficient HIIT training prescription in improving health outcomes for such a population.

The current study has several limitations. First, the pre- and post-measures for some subjects were taken in the follicular phase, while others in late luteal phase, instead of all of them in the same phase of the menstrual cycle. Given the fact that the intervention was for 5 weeks only, it would have been unlikely to choose the same phase of the menstrual cycle among subjects of such a population. More importantly, the high rate of abnormal menstrual cycle in overweight and obese women (54% in the present study) suggested that the subjects might have PCOS, the most common endocrinopathy in young overweight/obese women [48]. Because we had no diagnosis of this disease, we were not able to assess the potential effect of this condition. Second, as a common tool to assess body weight and the relevant parameters of body composition, bioelectrical impedance analysis was used in the present study. However it is not the “gold standard” in body composition measurement. Considering the efficacy of aerobic high-intensity exercise in reducing visceral fat, and the effectiveness of HIIT for reducing trunk fat [3], more accurate techniques, for instance, Dual-energy X-ray absorptiometry (DEXA), magnetic resonance imaging (MRI), or computed tomography (CT), could be used in future studies. These more sophisticated techniques can provide more detailed information about body composition, such as estimation for adipose tissue and muscles, and the information about the distribution pattern of adipose tissue on different body parts (trunk, visceral, subcutaneous, and intermuscular depots). Third, given that various HIIT protocols may have different effects on weight loss in females, issues such as acute responses to high-intensity intermittent exercise and potential effect of HIIT on sex hormones need to be examined in future studies for such a population (i.e., overweight and obese women). Finally, this study does not provide information on a host of other potentially relevant factors (e.g., nutrition status, exercise modality, daily activity, fitness level, weight change, menstrual cycle, etc.) that may affect this population (i.e., overweight and obese women) on the parameters studied in the present study. Future studies may consider tighter control of these factors such that the effects of these different factors could be isolated and identified in a relatively longer intervention.

In conclusion, the present study shows that when compared to MVCT, the HIIT exercise protocol implemented in this study is a more enjoyable and time-efficient approach in improving cardiorespiratory fitness and ameliorating sexual hormones in obese young women. The mild HIIT protocol might be useful for maintaining stable body weight in the sedentary population.

## Supporting Information

S1 Checklist CONSORT Checklist Document. (DOC)

S1 Dataset Dataset Document. (XLSX)

S1 Protocol Exercise Training for Cardiorespiratory Fitness and Weight Loss in Obese Young Women. (PDF)
