# Supplementary material for: Comparison of High-Intensity Interval Training and Moderate-to-Vigorous Continuous Training for Cardiometabolic Health and Exercise Enjoyment in Obese Young Women: A Randomized Controlled Trial
Source: PLoS One. 2016 Jul 1;11(7):e0158589. doi: 10.1371/journal.pone.0158589 (PMC4930190; doi:10.1371/journal.pone.0158589)
Supplement: S1 Protocol — (PDF) [file pone.0158589.s003.pdf]

Exercise Training for Cardiorespiratory Fitness and Weight Loss in Obese Young Women[1]

Principal Investigator

Dr. Kong Zhaowei
Faculty of Education, University of Macau

[1] Supplement Experiment for Effects of Hypoxic Exercise on Weight Loss and Heart Protection in Obese Adults (MYRG027(Y1-L1)-FED11-KZW)

ABSTRACT OF THE PROJECT (Max. 200 words)

A growing body of evidence suggests that high-intensity interval training (HIIT) improves aerobic fitness and body composition that are similar to or better than training endurance training eg. moderate-to-vigorous intensity training (MVCT) despite a lower training time commitment. However, for sedentary individuals, the strategy that HIIT as an alternative to traditional continues exercise has been argued because it is extremely hard. Although it has been reported that HIIT seems more enjoyable than MVCT, such findings were from young active men or normal weight individuals. Whether HIIT results in superior improvements in cardiorespiratory fitness and metabolic indicators when compared with MVCT in obese individuals warrant further investigation. In terms of the study designs for comparing fat loss between HIIT and MVCT, there are common flaws including lacking quantitative estimation of energy expenditure and neglecting the effects of additional physical activities besides the exercise training program. Thus, whether short-term HIIT training is superior to MVCT for improving body composition in overweight and obese individuals has not been fully supported. In addition, overweight and obesity may affect women's health with irregular menstrual cycle and abnormal sex hormones, thus it is necessary to examine whether HIIT has influences on sex steroid hormones in sedentary overweight young women.

PROJECT DETAILS

Objectives

Using a less demanding HIIT protocol with 60 repetitions of 8s/12s work-rest intervals at a lower resistance for 4 days/week, the purpose of this study was to compare the effects of 5-week HIIT and MVCT interventions on cardio-metabolic health outcomes, sex hormones levels and physical activity enjoyment scale (PAES) in overweight female subjects.

Background of the Project

Overweight and obesity, associated with a number of comorbidities and metabolic disorder, has become a serious public health problem worldwide. Weight reduction improves cardiorespiratory fitness and lowers metabolic risk factors [Garber et al, 2011]. Although weight increase occurs for all ages in both developed and developing countries, such increase is the most rapid for people between 20 and 40 years old [Ng et al, 2014].

The recommended exercise prescription for most adults is typically a regular moderate-intensity continuous exercise program for a total of over than 150 min·wk^{-1} to improve and maintain physical fitness and health [Garber et al, 2011]. However, the inactive nature of sedentary individuals and the "lack of time" excuse tend to prevent people from being engaged in regular physical activity [Hardcastle et al, 2013].

An increasing amount of evidence indicates that high-intensity interval exercise (HIIT) has the potential to improve cardiorespiratory fitness, thus reducing obesity and the associated comorbidities [Trapp et al, 2008; Perry et al, 2008]. However, although previous studies have reported that 2-week HIIT protocol resulted in marked

increments in $\dot{V}O_{2max}$ by 9% to 13%, the improvement of cardiorespiratory fitness as a result of HIIT did not always happen in 2 to 6 weeks of HIIT training [Iaia et al, 2009; Astorino et al, 2011; Allemeier et al, 1994]. At present, in the studies that compared continuous exercise with HIIT, not a few have observed the physiological differences between MVCT training around 150 minutes per week and HIIT training.

Overweight and obesity may affect women's health with irregular menstrual cycle and abnormal sex hormone levels [Kulie et al, 2011], as high levels of androgen and estradiol are observed in obese young women [Tchernof & Despres, 2000]. Sex steroids hormones are associated with the regulation of body fat distribution in both men and women [Tchernof & Despres, 2000], therefore, sex steroid hormones may play a supportive role in changes of body weight. However, to our knowledge, no researches have reported whether HIIT training has influences on sex hormones in sedentary overweight young women.

It has been reported that training with 8s/12s work-rest intervals in 20 minutes for 15 weeks improved body fat distribution and insulin resistance of young women, when compared to 40 min of steady-state exercise with the similar energy expenditure as HIIT [Trapp et al, 2008]. Given that the study designs for comparing fat loss between HIIT and MVCT has the common flaws, such as the lack quantitative estimation of energy expenditure [Lunt et al, 2014], and neglecting the effects of additional physical activities of the participants besides the implemented exercise training program [Trapp et al, 2014]. Thus, whether short-term HIIT training is superior to MVCT for weight loss and body fatness reduction in overweight and obese individuals has not been fully supported.

Recently, there has been the argument against the use of HIIT as an alternative to

traditional continuous exercise, because HIIT training is extremely hard; as a result, it is likely to be a deterrent for the largely sedentary/obese population [Ekkekakis et al, 2011; Hardcastle et al, 2013]. Enjoyment is an important factor for long-term adherence. Although it has been reported that HIIT seems more enjoyable than either MVCT [Bartlett et al, 2011] or continuous vigorous-intensity exercise [Jung et al, 2014], such findings were from young active men [Bartlett et al, 2011] or normal weight individuals [Jung et al, 2014]. Because of these considerations, the claim that low-volume HIIT training is more enjoyable than MVCT training needs to be examined further in different populations.

Based on the above knowledge, it is hypothesized that: 1) both HIIT and MVCT would induce improvements in cardio-metabolic parameters including $\dot{V}O_{2max}$, body weight and fat, blood lipids, and sexual hormones, 2) the time-efficient HIIT would be a more enjoyable exercise mode in comparison with MVCT.

Research Methodology and Implementation Plan

Participants

A public notice including the research purpose, qualifying criteria, research process, matters need attention and privacy protection will be released to the participants who are interested in this study. The inclusion criteria for the subjects are as follows: (1) between 18 and 30 years old; (2) sedentary as defined by reporting less than 60 minutes exercise every week in the previous 6 months; (3) body fatness over than 30% measured by a 5-serial of frequent bioimpedance analyzer (Biospace Inbody 720, South Korea), and (4) a doctor certificate approving the practice of vigorous exercise. Each volunteer needs to complete a PAR-Q form and a menstrual cycle survey prior to being admitted into the study. The subjects who have any barriers to

physical activity, smokers, alcoholics, and contraceptive or prescribed drug users will be excluded.

With a power of 0.8 at $\alpha = 0.05$, an assumed correlation of 0.8 between pre-treatment and post-treatment, the sample size for HIIT group is estimated to be 12. When a dropout rate of 20% is allowed, a total sample size of 30 subjects is needed. 30-36 qualified sedentary overweight young females will be recruited. All subjects should provide written informed consent before participation. The subjects will be randomly assigned to either HIIT or MVCT group. By the end of the study, 80% subjects are expected to complete the experiment (Fig 1).

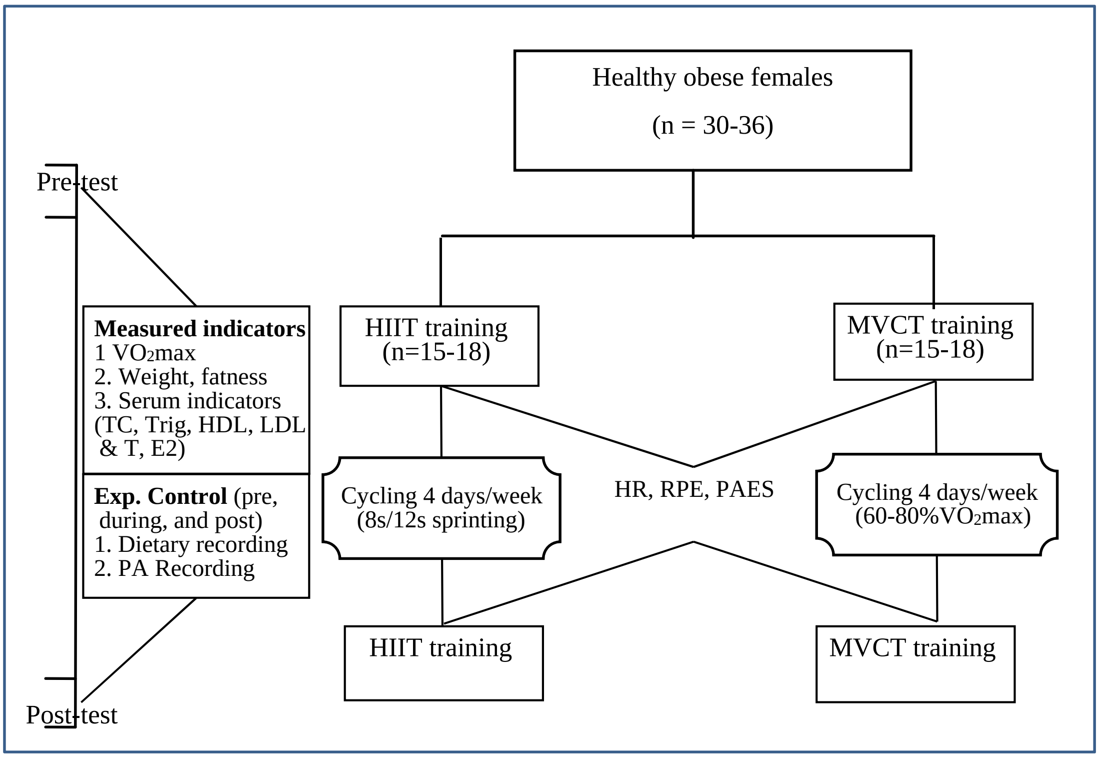

Fig 1 Manipulation Route

Experimental protocol

The experimental protocol consists of pre- and post-training measurements and a 5-week HIIT or MVCT intervention. Measures of $\dot{V}O_{2max}$, body composition and blood assays be taken in pre- and post-training measurements. Baseline measures will be

taken within 3-7 days before the 5-week exercise intervention, and post-training measures will be taken 3-7 days following the last training session. All pre- and post-training testing sessions will be arranged in the follicular or late luteal phase of each subject according to the self-reported menstrual cycle survey. During test and training sessions, the same verbal encouragements will be given by the same helpers.

Exercise training intervention

The HIIT cycling protocol is from a previous study done by Trapp et al. (2008). After a 3-min warm-up at 50 W, each participant will conduct 8 s of sprinting and 12 s of passive rest for a maximum of 60 repetitions on an ergometer. The initial resistance is 0.5 kg and the resistance will be increased by 0.5 kg once an individual can complete two consecutive 20-min intermittent sprinting exercise sessions at the given workload. Subjects will have a cool down for 3 min at 50 W followed by standard stretches. Heart rate, ratings of perceived exertion (RPE) and training power will be recorded before and after every five sprints. Enjoyment of exercise will be assessed by PAES scale [Kendzierski & DeCarlo, 1991] immediately after every training session. When an individual misses a session, she should make it up later in order to complete 20 HIIT sessions within 5 weeks. To estimate exercise intensity and energy output, $\dot{V}O_2$ measures during the first and last training session will be taken for all the subjects in HIIT group.

For MVCT training [Trapp et al, 2008], each subject starts a 3-min warm-up at 50 W, and then exercises at an initial workload of 60% $\dot{V}O_{2max}$ of the pre-training test for 40 min with a rhythm at 60 rpm. After every training session, a 3-min cool-down and stretching will be conducted. Once an individual can complete two consecutive exercise sessions at the specified level of exercise strength, resistance will be

increased by 0.5 kg until the workload being reached the level of 80% $\dot{V}O_{2max}$ of the pre-training test. HR, RPE and training power will be recorded every 5 min during the exercise training, PAES scores will be assessed in the same way as for the HIIT group.

Measures

Body composition

Height and weight will be measured by using standard conventional methods with light clothing. Body mass index (BMI) was calculated by weight and height. Body composition will be assessed after baseline blood sampling and 3 days following the last training session.

$\dot{V}O_{2max}$ test

$\dot{V}O_{2max}$ will be performed at baseline and at least 3 days following the last training session. After a 2 min cycling warm-up at 30 W, graded exercise test will start at the initial workload of 50 W and increased by 25 W every 3 min till completion. Subjects will be asked to maintain a cycling speed of 60 rpm unless reaching the criteria or volitional exhaustion. $\dot{V}O_{2max}$ will be assessed by a calibrated breath-by-breath analysis system (Meta-Max 3B, Cortex Biophysik GmbH, Leipzig, Germany). The test will be terminated when the subjects meet any two of the following criteria: (1) $\dot{V}O_2$ reached a plateau with a change less than 150 $mL\cdot min^{-1}$), (2) heart rate reached age-predicted maximal level (220-age), (3) respiratory exchange ratio (RER) $\geqslant$ 1.10, and (4) rating of perceived exertion (Borg's) scale reached. A 5-min recovery at 30 W will be carried out followed by the test.

Blood lipids and sexual hormones

Blood samples will be taken from the cubital veins at pre- and post-training after a minimum of a 12 h overnight fast. After being clotted for 1 hour at room

temperature, the samples will be centrifuged at 3000 rpm for 10 min and then serum samples will be prepared and stored at a low-temperature refrigerator. High-density lipoprotein cholesterol (HDL-C), low-density lipoprotein cholesterol (LDL-C), total cholesterol (TC) and total triglyceride (TG) and sexual hormones including testosterone and estradiol will be analyzed in the same assay.

Diet and extra physical activity

Subjects will be instructed to maintain their normal diet and normal daily physical activities throughout the intervention. Food intake data will be recorded by using a 3-day diet recall protocol (two weekdays and one weekend day) during three weeks: one week before the intervention, the third week of the intervention, and the last week of the intervention. Energy intake and diet components will be analyzed by nutrition analysis and management system (Sports Nutrition Research Center, National Institute of Sports Medicine, China). Daily physical activities will be monitored by wearing a pedometer (Yamax SW-200 digiwalker, Japan) on three days in a week (two weekdays and one weekend day) in every week during the 5-week training period.

Statistical analysis

A two-way mixed analysis of variance (ANOVA) with repeated measures will be used to test the main effect (i.e., group effect) and the interaction effect (time and group interaction) on the outcome variables. For within–group change from pre- and post-values of the relevant outcome variables of interest, paired-sample *t*-test will be performed to test the difference in pre- and post-measures. Change between the pre- and post-measures will be calculated for each outcome variable of interest. Independent-sample *t*-test will be performed to determine the differences of the change and of the percentage change between the two groups. Pearson-product

moment correlation coefficients will be conducted to examine the relationships between the cardio-metabolic health outcomes and sexual hormones.

REFERENCES

Allemeier CA, Fry AC, Johnson P, Hikida RS, Hagerman FC, Staron RS (1994). Effects of sprint cycle training on human skeletal muscle*. J Appl Physiol*, 77(5): 2385-2390.

Astorino TA, Allen RP, Roberson DW, Jurancich M, Lewis R, McCarthy K, Trost E (2011). Adaptations to high-intensity training are independent of gender. *Eur J Appl Physiol,* 111: 1279–1286.

Bartlett JD, Close GL, MacLaren DPM, Gregson W, Drust B, Morton JP (2011). High intensity interval running is perceived to be more enjoyable than moderate-intensity continuous exercise: implications for exercise adherence. *J. Sport Sci,* 29: 547–559.

Campbell SE, Febbraio MA (2001). Effect of ovarian hormones on mitochondrial enzyme activity in the fat oxidation pathway of skeletal muscle. *Am J Physiol Endocrinol Metab,* 281: E803-808.

Durstine JL, Grandjean PW, Davis PG, Ferguson MA, Alderson NL, DuBose KD (2001). Blood lipid and lipoprotein adaptations to exercise: a quantitative analysis. *Sports Med*, 31(15): 1033-1062.

Ekkekakis P, Parfitt G, Petruzzello SJ (2011). The pleasure and displeasure people feel when they exercise at different intensities: Decennial update and progress towards a tripartite rationale for exercise intensity prescription. *Sports Med*, 41 (8): 641-671.

Garber, Carol Ewing, Blissmer, Bryan, Deschenes, Michael R, Franklin, Barry A, Lamonte, Michael J, Lee, I-Min M.D., Sc.D, Nieman, David C, Swain, David P (2011). Quantity and Quality of Exercise for Developing and Maintaining Cardiorespiratory, Musculoskeletal, and Neuromotor Fitness in Apparently Healthy Adults: Guidance for Prescribing Exercise. *Medicine & Science in Sports & Exercise,* 43(7): 1334-1359.

Hardcastle SJ, Ray H , Beale L, Hagger MS (2013). Why sprint interval training is inappropriate for a largely sedentary population. *Frontiers in Psychology,* 11 (23): 1037–1042.

Iaia FM, Hellsten Y, Nielsen JJ, Fernström M, Sahlin K, Bangsbo J (2009). Four weeks of speed endurance training reduces energy expenditure during exercise and maintains muscle oxidative capacity despite a reduction in training volume. *J Appl Physiol,* 106(1): 73–80.

Jung ME, Bourne JE, Little JP (2014). Where does HIT fit? An examination of the affective response to high-intensity intervals in comparison to continuous moderate- and continuous vigorous-intensity exercise in the exercise intensity-affect continuum. *PLoS One,* 9(12): e114541. doi: 10.1371/ journal. pone. 0114541.

Kendzierski D, DeCarlo KJ (1991). Physical Activity Enjoyment Scale: Two Validation Studies.

Journal of Sport & Exercise Psychology, 13(1): 50-64.

Kulie T, Slattengren A, Redmer J, Counts H, Eglash A, Schrager S (2011). Obesity and women's health: an evidence-based review. *J Am Board Fam Med*, 24(1): 75-85.

Lunt H, Draper N, Marshall HC, Logan FJ, Hamlin MJ, Shearman JP, Cotter JD, Kimber NE, Blackwell G, Frampton CM (2014). High intensity interval training in a real world setting: a randomized controlled feasibility study in overweight inactive adults, measuring change in maximal oxygen uptake. PLoS One, 9(1): e83256.

Ng M, Fleming T, Robinson M, et al. (2014). Global, regional, and national prevalence of overweight and obesity in children and adults during 1980–2013: a systematic analysis for the global burden of disease study 2013. *Lancet*, 384: 766–781.

Perry CGR, Heigenhauser GJF, Bonen A, Spriet LL (2008). High-intensity aerobic interval training increases fat and carbohydrate metabolic capacities in human skeletal muscle. *Applied Physiology, Nutrition and Metabolism,* 33(6): 1112–1123.

Talanian JL, Galloway SDR, Heigenhauser GJF, Bonen A, Spriet LL (2007). Two weeks of high-intensity aerobic interval training increases the capacity for fat oxidation during exercise in women. *Journal of Applied Physiology*, 102(4): 1439–1447.

Tchernof A, Despres JP (2000). Sex steroid hormones, sex hormone-binding globulin, and obesity in men and women, *Horm Metab Res.,* 32 (11–12): 526–536.

Trapp E, Chisholm D, Freund J, Boutcher S (2008). The effects of high-intensity intermittent exercise training on fat loss and fasting insulin levels of young women. *Int J Obes*, 32: 684-691.

FED	PROJECT REF	PROJECT TITLE	PI	Panel's Comments/Remarks	Further Supporting Docs Required	Assessment Result
1	MYRG027(Y1-L1)-FED11-KZW<br><br>(Supplementary)	EFFECTS OF HYPOXIC EXERCISE ON WEIGHT LOSS AND HEART PROTECTION IN OBESE ADULTS	Kong Zhaowei	PI has provided all documents required for supplementary assessment and is satisfactory	N/A	**APPROVED**
